# Introducing the E‐MOTIVE Bundle for the Management of Postpartum Hemorrhage: Perceptions of Health Professionals and Perceived Barriers to Its Implementation in Sub‐Saharan Africa

**DOI:** 10.1155/jp/4290694

**Published:** 2026-04-29

**Authors:** Wondimu Gudu, Felagot Tadesse, Abraham Fessehaye Sium, Aida Tilahun, Abraham Getachew

**Affiliations:** ^1^ Department of Obstetrics and Gynaecology, Saint Paul’s Hospital Millennium Medical College, Addis Ababa, Ethiopia, sphmmc.edu.et; ^2^ Research Directorate, Saint Paul’s Hospital Millennium Medical College, Addis Ababa, Ethiopia, sphmmc.edu.et

**Keywords:** barriers, E-MOTIVE bundle, Ethiopia, healthcare professionals, postpartum hemorrhage, PPH, qualitative study

## Abstract

**Background:**

The E‐MOTIVE bundle that includes objective measurement of early blood loss has been found to be effective in the early detection and management of PPH and the reduction of maternal mortality because of PPH. The aim of this study was to determine the perception of trained health professionals toward the E‐MOTIVE bundle intervention for PPH as well as to explore perceived barriers to its implementation in Ethiopia.

**Methods:**

This mixed‐methods study was conducted at Saint Paul’s Hospital in Addis Ababa, Ethiopia, from February 1, 2024, to May 31, 2024. Data were collected from 80 health professionals using a structured questionnaire for the quantitative component, and seven in‐depth interviews (IDIs) were conducted for the qualitative component with stakeholders working in maternal health. Quantitative data analysis was performed using SPSS Version 23.1, whereas qualitative data were analyzed thematically using ATLAS.ti Version 7.0.71. Written consent was obtained from study participants.

**Results:**

Only 21% of the health professionals had heard of E‐MOTIVE prior to the training. More than 90% of the health professionals and all of the participants of the IDIs believed that the E‐MOTIVE bundle comes with a novel approach for early detection of PPH and is feasible and acceptable. Most perceived that the E‐MOTIVE approach is easy to understand and motivating for staff. The major perceived barriers to the implementation of the bundle were as follows: the huge resource implications of the bundle (85% of respondents), the need for increased clinical engagement (66%), increased labor ward stays of mothers (35%), and the need for more clinical documentation (38%). Other barriers cited by IDI participants were as follows: the potential for creating a displeasing labor and delivery setup, unavailability of the blood collection drapes and challenges with their local production, concern about the cultural acceptability of the drapes, and the need to align with national guidelines on PPH management.

**Conclusions:**

The E‐MOTIVE bundle approach for PPH management is feasible and acceptable. However, its successful implementation hinges on coordinated efforts to incorporate it into guidelines, overcome resource limitations, and implement behavior change strategies targeted toward healthcare professionals and delivering mothers.

## 1. Introduction

Postpartum hemorrhage (PPH) is the leading cause of preventable maternal death [1]. The definition of PPH has been updated in the recent consolidated guidelines for the prevention, diagnosis, and treatment of PPH. Detection is based on a measured blood loss of ≥ 300 mL accompanied by abnormal vital signs, such as tachycardia or hypotension, and/or when a blood loss threshold of ≥ 500 mL is reached [2]. Early identification of bleeding and prompt management using evidence‐based guidelines can avert most PPH‐related severe morbidities and deaths [3]. In a significant breakthrough in the management of PPH, the E‐MOTIVE trial found that a multifaceted health service intervention reduced severe PPH after vaginal delivery by 60% in 78 hospitals in Nigeria, Kenya, Tanzania, and South Africa. The E‐MOTIVE approach comprises objective blood loss measurement monitored every 15 min during the first hour after delivery to detect PPH early and trigger a bundle of first‐line treatments, including massaging the uterus, oxytocin infusion, tranexamic acid infusion, intravenous crystalloid fluids, examination for the cause, emptying the bladder, and, if necessary, escalation of care [4]. This breakthrough intervention provides hope for women in low‐income countries, including sub‐Saharan Africa, which share the largest percentage of maternal mortality globally and where PPH is a top cause of maternal death. However, its implementation has been hindered for several reasons, among which are negative health professional attitudes and wrong perceptions about the practice, among other perceived barriers [5]. Hence, there exists a critical need to address context‐specific barriers to the early and timely detection and management of PPH using the E‐MOTIVE approach. A qualitative study conducted in Kenya, South Africa, and Nigeria identified four key themes, which are as follows: in‐service training on obstetric care, limited knowledge about PPH, current approaches to PPH, and current PPH management and associated challenges [6].

Ethiopia has made tremendous improvements in maternal health with a significant reduction in maternal mortality, but PPH still remains the major driver of maternal mortality. Although the E‐MOTIVE bundle has been shown to be an effective approach to reduce severe postpartum hemorrhage, this intervention is not well incorporated into clinical practice in Ethiopia. Recently, however, there have been ongoing efforts to introduce the E‐MOTIVE bundle into the management of PPH, and it is already being piloted at Saint Paul’s Hospital, one of the largest referral hospitals in Ethiopia. The first 120 maternal healthcare providers have been trained on E‐MOTIVE. Hence, our study is aimed at assessing the perception of these professionals toward E‐MOTIVE and exploring perceived barriers to the implementation of E‐MOTIVE through in‐depth interviews (IDIs) with healthcare professionals and selected program managers employed at organizations working in maternal health.

## 2. Materials and Methods

This was a mixed‐methods study (quantitative, descriptive survey and qualitative phenomenology) conducted at Saint Paul’s Hospital Millennium Medical College, Ethiopia, from February 1, 2024, to May 31, 2024. The hospital is one of the highest referral centers in the country. It provides comprehensive emergency obstetric care with an estimated 10,000 annual deliveries.

The study participants for the quantitative study were healthcare professionals working at labor and delivery units of Saint Paul’s Hospital who were trained on the E‐MOTIVE bundle approach for the management of PPH. The participants for the qualitative study were maternal healthcare providers and other nonclinical professionals working in organizations engaged in maternal health. Maximum variation sampling was used to represent diverse health professionals with different levels of roles and professional experiences. The sample size for the quantitative data was determined by convenience, and all healthcare professionals who were trained on E‐MOTIVE and willing to participate were recruited. For the IDI, a minimum of 5 and a maximum of 10 IDIs were planned to be conducted, but the total sample size was determined by information saturation.

The quantitative data were collected using a self‐administered questionnaire, prepared in English. The contents of the questionnaire included information on sociodemographic characteristics and the professional profile of participants and 20 perception questions. Data were collected by a trained midwife. Qualitative data were collected through IDIs with midwives, residents, obstetricians and gynecologists, and stakeholders from professional associations, funding bodies, program designers, and implementers. The IDIs were conducted by general medical practitioners experienced in qualitative data collection. An interview guide was prepared, and questions were set after an extensive review of the literature and based on experience from clinical practice. Further recruitment of participants was stopped when thematic saturation was reached. The principal investigator made frequent checks on the data collection process to ensure the completeness and consistency of the gathered information.

Quantitative data were analyzed using SPSS Version 23, and simple descriptive statistics were used. Data were presented in tables. All IDIs were transcribed verbatim, and qualitative data analysis was conducted using ATLAS.ti Version 7.0.71. Sixty‐seven codes and five families were generated from the analysis process and were later considered themes.

A formal letter of approval was obtained from the IRB at SPHMMC (Ref. # Pm23/407). Verbal consent was obtained from study participants for both the quantitative and qualitative studies.

## 3. Results

### 3.1. Sociodemographic and Professional Profile of the Study Participants

There were 80 maternal healthcare professionals who participated in the quantitative component of this study. Most (67%) were male and single (75%). The participants included gynecologists, residents, midwives, and clinical nurses. Equal (50%) proportions of the participants were physicians/residents and midwives/nurses (Table 1). Seventy‐five percent of the professionals had work experience of less than 5 years. Among the nurses, most (49%) had degrees (MSc/BSc). Most (99%) of the study participants had previous experience managing patients with PPH. Only 17 (21%) of the health professionals had previously heard of the E‐MOTIVE bundle approach for the management of PPH (Table 1), and their primary sources of information (41%) were seniors/consultants practicing at health facilities.

**Table 1:** Perception of healthcare professionals toward the E‐MOTIVE bundle approach to the management of PPH at Saint Paul’s Hospital, Ethiopia, 2024.

S.No.	Description	Strongly disagree (1)	Disagree (2)	Neutral (3)	Agree (4)	Strongly agree (5)
1	Helps detect more cases compared to the traditional method	1 (1.3)			15 (18.8)	64 (80)
2	Novel approach of PPH detection		3 (3.8)	4 (5)	33 (41.3)	40 (50)
3	Novel approach of PPH management	10 (12.5)		9 (11.3)	33 (41.3)	28 (35.0)
4	Easily understandable approach		1 (1.3)	3 (3.8)	28 (35)	48 (60)
5	Easily accepted by all staff		1 (1.3)	12 (15)	35 (43.8)	32 (40)
6	More motivating for staff compared with existing practice	1 (1.3)	2 (2.5)	8 (10)	36 (45)	33 (41.3)
7	Organized, understandable algorithms			4 (5)	34 (42.5)	42 (52.5)
8	Objective assessment of vaginal bleeding	1 (1.3)		2 (2.5)	24 (30)	53 (66.3)
9	Decreases maternal mortality because of PPH			4 (5)	30 (37.5)	46 (57.5)
10	Has insignificant resource implications	29 (36.3)	39 (48.8)	10 (12.5)	1 (1.3)	1 (1.3)
11	Needs the same engagement of healthcare workers	25 (31.3)	28 (35)	13 (16.3)	12 (15)	2 (2.5)
12	Will not increase labor ward stays of mothers	11 (13.8)	17 (21.3)	10 (12.5)	31 (38.8)	11 (13.8)
13	Takes the same time as the routine management approach	8 (10)	16 (20)	8 (10)	30 (37.5)	18 (22.5)
14	Overtreatment of PPH unlikely	9 (11.3)	20 (25)	8 (10)	29 (36.3)	14 (17.5)
15	Documentation not time‐consuming	6 (7.5)	24 (30)	14 (17.5)	25 (31.3)	11 (13.8)
16	Blood collection bags not discomforting to mothers	4 (5)	21 (26.3)	20 (25)	22 (27.5)	13 (16.3)
17	Culturally acceptable drapes	3 (3.8)	12 (15)	24 (30)	29 (36.3)	12 (15)
18	Recommends rolling out E‐MOTIVE nationally	2 (2.5)	3 (3.8)	9 (11.3)	38 (47.5)	28 (35)
19	Overall makes detection and management easier	11 (13.8)	18 (22.5)	14 (17.5)	22 (27.5)	15 (18.8)
20	Can be implemented at all levels of health facilities	8 (10)	2 (2.5)	19 (23.8)	n (33.8)	24 (30)

*Note:* Five‐point scale, from strongly disagree to strongly agree; *n* = 80; scale, number (%).

Among the seven IDIs conducted, three were with healthcare professionals practicing at Saint Paul’s Hospital. These included a midwife, a resident, and an obstetrician and gynecologist who were working at the labor and delivery wards. The other four participants were stakeholders from professional associations: the presidents of the Ethiopian Society of Obstetricians and Gynecologists and the Ethiopian Midwives Association, program managers from Jhpiego and BMGF, and the coordinator of the maternal health directorate at the Ministry of Health (MOH). All of these participants had heard about the E‐MOTIVE bundle and had the chance to participate in meetings, trainings, and international conferences, which enabled them to assume their current roles as project designers and writers, funders, and participants as guideline developers representing the MOH, professional associations, or funding agencies.

### 3.2. Perception of Healthcare Professionals Toward the E‐MOTIVE Bundle Approach to the Management of PPH

The results of the quantitative study on the perception of healthcare providers toward E‐MOTIVE are shown in Tables 1 and 2. Considering the mean as a cutoff point to dichotomize the responses of participants, around half (51.0%) of the study participants had a negative perception toward the E‐MOTIVE bundle for the management of PPH.

**Table 2:** Perception of healthcare professionals toward E‐MOTIVE bundle intervention at Saint Paul’s Hospital Millennium Medical College.

	Frequency	Percent	Cumulative percent
Negative perception	43	53.8	53.8
Positive perception	37	46.3	100.0
Total	80	100.0	

In the qualitative data analysis, 67 codes and 5 families were generated, and these were later considered themes. Findings from the quantitative study and the IDIs were triangulated and presented under the following five themes.

#### 3.2.1. Theme I: Changes in Existing Practice in the Management of PPH

Almost all of the healthcare professionals (99%) believed that the E‐MOTIVE bundle for PPH management helps detect more cases compared to the traditional approach. Ninety‐one percent believed that the E‐MOTIVE bundle comes with a novel approach to detection (91%) and management (76%) of PPH. The major change reported by 96% of the participants was the objective assessment of vaginal bleeding. The other changes reported were as follows: well‐organized, understandable algorithms (95%) and more motivating for staff compared to existing practice (86%).

The IDI respondents signposted the change as the introduction of calibrated drapes for objective measurement of bleeding as a clear‐cut definition of PPH, which they mentioned as unique. However, the majority agreed that the components of the approach are identical to the existing PPH management routine approaches (*n* = 5). The proposed bundle is believed to improve the detection of more PPH cases that would have been missed by the traditional method of estimating blood loss. The drapes avoid subjective measurements of blood loss that were variably reported by healthcare providers.

Most of the things exist and were used previously. The new thing is the drape to estimate the blood. Normally it was the challenge to estimate the blood when we work as professional at health centers because amniotic fluid and urine were mixed. Therefore, that was the challenge to estimate. (Participant 2)

#### 3.2.2. Theme II: Enablers and Opportunities

Alignment of E‐MOTIVE with national guidelines, the piloted approach at different healthcare systems in the African region and beyond, the possibility of local drape production, the presence of a policy platform including safe motherhood, a supportive RH strategy that targets alleviating maternal mortality, stakeholders’ interest, and the involvement of the MOH at the lead position were the repeatedly cited codes by the interview participants.

Strong evidence is generated when we see globally like as this bundle works. When we see according to WHO adoptions, these things are good. It creates an opportunity to adopt in ecosystem easily in Ethiopia. (Participant 3 Q14)

Correspondingly, most participants pointed out clients’ positive attitudes toward providers, improved awareness of clients about hemorrhage, and improved birth preparedness during antenatal care, and the existence of active professional associations and stakeholders that work on advocacy and awareness creation was stated as a potential opportunity for E‐MOTIVE implementation. On top of this, being built into the existing practice of PPH management can pose a possibility of integration.

When we see Ministry of Health and others, there is willingness. It is not only from the government side but also from the donor partner’s side. (Participant 3 Q3:16)

#### 3.2.3. Theme III: Perceived Barriers and Challenges

Possible challenges and perceived barriers to the implementation of PPH management expressed in the quantitative study by the healthcare professionals were as follows: the huge resource implications of the E‐MOTIVE bundle (85% of respondents), the need for increased clinical engagement (66%), increased labor ward stays of mothers (35%), and the need for more clinical documentation (38%) (Table 2).

Respondents of the IDIs also pointed out barriers to the effective implementation and future scale‐up of E‐MOTIVE. The overriding codes were issues of costs related to drapes, inconsistent supply of material and medication for the bundle, the requirement of production of drapes for the local market, and being an approach that requires stepwise activity.

… IV fluids may be sometimes not available, TXA may expire, and also the other resources may be insufficient. This is in tertiary hospital. As we go to health centers and rural areas the problems become more significant. (Participant Q3:21)

New drapes cost is an issue. Why? Because there are yearly dedicated commodities for maternal health in percent. When this comes, it is an additional. (Participant Q2:30)

The first challenge I think, is the cost. Because finding these calibrated drapes is difficult currently even for trial purpose because of their cost. (Participant 4 Q4:14)

Likewise, most respondents stated that future challenges that might be encountered include the existence of overwhelmed services, issues of waste management because of newly generated drape waste, the requirement of guideline revision, and local evidence that shows context‐specific implementation follow‐up.

The patients number is a lot, there might not be ways to manage aggressively like in its minimum threshold as per E‐MOTIVE’s saying. Many times as there is repetitive overflow of patients, it may be one challenge. (Participant 1 Q1:24)

Some of the other concerns were as follows: the potential of creating a displeasing labor and delivery setup, the addition of a new burden to the system, reduced staff motivation, shortages of allocated maternal and child health budget, difficulties in licensing drapes as a medical item, and shortages of foreign exchange if importing drapes is used as a means of supply in the system, and the requirement of proper documentation practices.

#### 3.2.4. Theme IV: Acceptability of the E‐MOTIVE Bundle and the Blood Collection Drape

Among the participants of the quantitative study, 95% believed that the E‐MOTIVE bundle approach is easily understandable, and 84% thought it is easily acceptable by all levels of maternal healthcare providers. Most (64%) believed that it can be implemented at all levels of health facilities, and 83% recommended rolling it out nationally. One of the concerns with E‐MOTIVE implementation is the acceptability of the blood collection drapes by the mothers. Overall, half of the healthcare professionals believed that the drapes are culturally acceptable. Although most (44%) of the healthcare professionals believed that the drapes are not discomforting to mothers (during and/or after application), 31% thought they are discomforting.

Most of the participants of the IDIs agreed that the E‐MOTIVE procedure is both feasible and acceptable. They described it as a noninvasive technique that clients are unlikely to resist. The intervention is considered doable and aligns well with existing practices for managing PPH.

… from the community it may not be big challenge because our community is not impulsive even for the paternal services in governmental health institutions. If we think it is important for them and if the health workers explain to them, they have no problem to support …. (Participant 1 Q1:11)

However, participants agreed that the acceptability of the E‐MOTIVE procedure requires groundwork and follow‐ups, including client awareness and hands‐on training for providers.

On the other hand, some participants viewed the intervention as a new burden, citing concerns over increased workload (Participants 1 and 2), the need for extra human resources, and waste management planning. Cultural acceptability varied, with some participants believing it would be acceptable if clients are well informed, while others thought it should be assessed through practice. Overall, respondents agreed on a stepwise approach of E‐MOTIVE, which integrates with existing practices and shifts from subjective to objective measurement of PPH using calibrated drapes, thereby enhancing better detection.

#### 3.2.5. Theme V: Remedies for Threats (Implementation Strategies)

The reflection from interviews revealed that E‐MOTIVE is not a one‐step task but rather a stepwise approach of follow‐up and monitoring of practice and supplies. Necessary preparations before large‐scale implementation, development of appropriate documentation to track the effects of improved maternal outcomes with evidence generation, and engagement of stakeholders in an unceasing way in advocacy and guideline revision to include the E‐MOTIVE bundle and the encouragement of local production of drapes were suggested to mitigate supply issues and overcome challenges and barriers of E‐MOTIVE implementation.

## 4. Discussion

This is the first study on the perception of health professionals and perceived barriers to E‐MOTIVE implementation in Ethiopia. The results of our study are believed to provide key entry points to address actual barriers to the effective implementation of the E‐MOTIVE bundle approach for the management of PPH that is going to be introduced into clinical practice in the country. Beyond local evidence generation and informing clinical practice, the findings will provide valuable inputs for initiatives/efforts to introduce the E‐MOTIVE bundle into maternal healthcare services in similar low‐ and middle‐income countries (LMICs).

Only 21% of the health professionals had heard of E‐MOTIVE in this tertiary referral hospital. This is despite the 2018 WHO guideline on the bundle approach of PPH management and the presence of revised national guidelines on PPH that incorporated the bundle approach to PPH management. A study conducted in three African countries on E‐MOTIVE also found poor knowledge of health professionals [5]. This implies that efforts should be escalated to improve awareness among maternal healthcare providers of the E‐MOTIVE bundle and the need for provision of regular in‐service training.

The major positive perception the healthcare providers had toward the E‐MOTIVE bundle was regarding the detection of PPH. The blood collection bag was believed to be the most important innovation in the management of PPH by 96% of the healthcare professionals. The implication of this is even more pronounced as research studies have shown that PPH detection is very low in LMICs [7]. The E‐MOTIVE trial that was conducted in five LMICs reported a 93% increase in the detection of PPH with the introduction of the drapes for blood loss estimation [4]. The fact that the overall approach of E‐MOTIVE was considered motivating for most staff, with the algorithms being easily understandable, will present a good opportunity for a relatively easier introduction of the bundle into clinical practice.

There are numerous barriers and challenges identified by the study participants for the effective implementation of the E‐MOTIVE bundle. Among these, the major one was the resource implications of the E‐MOTIVE bundle (by 85% of respondents). This was also a concern of health professionals involved in a qualitative study conducted in three African countries [6]. The remedy needs the engagement of health managers and stakeholders of the supply chain so that they ensure appropriate budgeting for PPH commodities and their uninterrupted supply. The availability and cost of the drapes were also a significant concern to all study participants. This will also be one of the major challenges for the effective implementation of the E‐MOTIVE bundle in other LMICs as well. To alleviate this, incorporating the principles of appropriate technology and engaging local bioengineers for the production of the drapes are a way out.

To address barriers related to the attitude and perception of health professionals toward E‐MOTIVE (which included acceptability, increased workload, and lower staff motivation) and the health system in general, effective behavioral change interventions should be put in place. It is known that identifying effective ways of encouraging healthcare providers to change their clinical practice and follow evidence‐based recommendations remains challenging [8] and that the dissemination of evidence‐based recommendations alone does not ensure implementation by healthcare providers [8, 9]. Failure to integrate new recommendations into clinical practice can be affected by the challenges of changing well‐established individual clinical behavior, as well as changing hospital protocols and healthcare systems [10]. The introduction of a new intervention needs a well‐planned strategy to influence the behavioral change of healthcare professionals. One of the strategies is incorporating models of behavior change into implementation research that should be initiated with the introduction of the E‐MOTIVE bundle [3].

An increase in labor ward stays of mothers expressed by 35% of the participants is one of the challenges, particularly at hospitals with a high volume of deliveries. Health administrators need to devise effective strategies to tackle this problem, which may need the restructuring of labor and delivery units.

The concerns expressed by both the participants of the qualitative and quantitative study on the acceptability of the drapes imply that the introduction of new interventions should consider the sociocultural acceptability of innovations that come with them. This is even more pronounced in sub‐Saharan Africa, where numerous sociocultural values influence the acceptability of medical innovations and medical care in general. This has also been identified as one factor influencing the implementation of the E‐MOTIVE bundle in a study conducted in three African countries [11, 12]. Hence, behavioral change communication targeting pregnant mothers is important to increase the acceptability of the application of the drapes after childbirth.

Overall, for the effective implementation of the E‐MOTIVE bundle for PPH, there should be concerted efforts involving all stakeholders at the policy, health system, health facility, and community levels to overcome barriers and exploit opportunities. Currently, there is an ongoing Cochrane review of qualitative studies aiming to describe and explore the perceptions and experiences of women, community members, lay health workers, and skilled healthcare providers, which is expected to come up with a collective, firm, and practice‐guiding conclusion on this perspective [12].

## 5. Conclusions

The findings of the current study revealed that the E‐MOTIVE bundle approach for PPH management is feasible and acceptable. However, its successful implementation hinges on coordinated efforts to overcome resource limitations and implement behavior change strategies targeted toward healthcare workers and delivering mothers. Being built on the existing PPH management protocol, the presence of a policy platform that advocates maternal mortality reduction, coupled with the commitment of the MOH and stakeholders for E‐MOTIVE implementation, and the overwhelming evidence of the effectiveness of the intervention from LMICs provide a potential opportunity to implement E‐MOTIVE at a large scale.

## Author Contributions

**Wondimu Gudu:** design, planning, conduct, data analysis, manuscript writing. **Abraham Getachew:** data collection and analysis. **Abraham Fessehaye:** design, proposal development. **Felagot Tadesse:** data collection, supervision. **Aida Tilahun:** data collection, supervision.

## Funding

No funding was received for this manuscript.

## Conflicts of Interest

The authors declare no conflicts of interest.

## Data Availability

The data supporting the study results (dataset) are shared on https://figshare.com, 10.6084/m9.figshare.27640533, and 10.6084/m9.figshare.27640551.
